# Mapping the Minnesota living with heart failure questionnaire (MLHFQ) to EQ-5D-5L in patients with heart failure

**DOI:** 10.1186/s12955-020-01368-2

**Published:** 2020-04-29

**Authors:** Sanjeewa Kularatna, Sameera Senanayake, Gang Chen, William Parsonage

**Affiliations:** 1 Australian Centre for Health Service Innovation, School of Public Health and Social Work, Institute of Health and Biomedical Innovation, Queensland University of Technology, 60 Musk Avenue, Kelvin Grove, QLD, 4059 Australia; 2 Centre for Health Economics, Building H, Dandenong Rd, 900 Australia; 3 Monash University, Caulfield East, VIC 3145 Australia; 4 Royal Brisbane and Women’s Hospital, Butterfield St, Herston, QLD 4029 Australia

**Keywords:** Mapping, Minnesota Living with Heart Failure (MLHFQ), EQ-5D-5 L, Utility, Direct mapping, Indirect response mapping

## Abstract

**Background:**

Mapping algorithms can be used to convert scores from a non-preference based instrument to health state utilities. The objective of this study was to develop mapping algorithms which will enable the Minnesota Living with Heart Failure Questionnaire (MLHFQ) scores to be converted into EQ-5D-5L utility scores that can be used in heart failure related cost utility studies.

**Method:**

Patients diagnosed with heart failure were recruited from Australia. Mapping algorithms were developed using both direct and indirect response mapping approach. Three model specifications were considered to predict the EQ-5D-5 L utility score using MLHFQ total score (Model 1), MLHFQ domain scores (Model 2), or MLHFQ item scores (Model 3). Six regression techniques, each of which has the capability to cope with either skewness, heteroscedasticity, ceiling effects and/or the potential presence of outliers in the data set were used to identify the optimal mapping functions for each of the three models. Goodness-of-fit of the models were assessed using six indicators. In the absence of an external validation dataset, predictive performance of was assessed using three-fold cross validation method. In the indirect response mapping, EQ. 5D 5 L responses were predicted separately using the MLHFQ item scores using ordered logit model.

**Results:**

A total of 141 patients participated in the study. The lowest mean absolute error (MAE) was recorded from the multivariable fractional polynomials (MFP) model in all three-model specifications. Regarding the indirect response mapping, results showed that the performance was comparable with the direct mapping approach based on root mean squared error (RMSE) but was worse based on MAE.

**Conclusion:**

The MLHFQ can be mapped onto EQ-5D-5 L utilities with good predictive accuracy using both direct and indirect response mapping techniques. The reported mapping algorithms would facilitate calculation of health utility for economic evaluations related to heart failure.

## Introduction

Cardiovascular disease (CVD) is one of the leading causes of death in the developed countries [1]. Heart failure (HF) is the fastest growing CVD in the world which poses a significant global burden, affecting nearly 26 million people worldwide [2]. It is a chronic debilitating illness in which the symptoms worsen with progression of the disease. Disease progression is associated with significant impact to the physical and social wellbeing, increased hospitalization [3, 4] as well as increased mortality. It is estimated that over 61,000 (6.9 per 1000 person-years) Australians aged ≥ 45 years are diagnosed with clinically over HF every year. Heart failure accounts for an estimated 150,000 hospitalisations and over 1 million days in hospital per annum [5, 6] and poses a significant burden to the health budgets globally. It is estimated that 1–2% of total healthcare expenditures in Europe and North America is spent for the treatment of HF [7]. In Australia, the annual cost of managing HF in the community is approximately $900 million and nearly $2.7 billion when considering the additional cost of in-patient care [8].

Economic evaluation presents evidence to inform comparative decisions, particularly about value for money. It is used by the regulatory agencies of Australia, United Kingdom and Switzerland in their evaluations of the cost-effectiveness of new health interventions prior to funding. Cost-utility analysis (CUA) is one method of economic evaluation that has been used to inform resource allocation decisions [9]. In CUA, health benefits are usually measured by quality-adjusted life-years (QALYs).

QALYs incorporate both changes in life expectancy and quality of life in a single metric. Utility is the component of the QALY that accounts for the quality of life which is measured using generic multi-attribute utility instruments (MAUIs) such as the EQ-5D or the SF-6D [10]. However, evidence indicate that the disease specific quality of life instruments are superior to generic instruments (eg. EQ-5D), owing to their superior sensitivity to changes in quality of life [11–13]. However, most disease specific quality of life instruments are not preference-based and cannot directly generate the utilities. Non-preference base instruments are characterized by measuring, but not valuing health states. Currently there are no HF specific MAUIs available to estimate utility, thus generic instruments such as EQ-5D are widely used [14].

In this context, mapping algorithms are of importance, as they can convert scores from a disease specific instrument to utilities. Mapping algorithms have been successfully developed to many disease specific quality of life instruments including instruments related to cardio-vascular diseases [15, 16]. However, to the authors’ knowledge, currently there is no study on the development of a mapping algorithm for a heart failure specific study instrument. The main objective of this study was to develop mapping algorithms which will enable the Minnesota Living with Heart Failure Questionnaire (MLHFQ) scores to be converted into utility scores that can be used in the heart failure related cost utility studies.

## Methods

### Study design

Ambulatory patients were recruited from cardiology out-patient clinics at Royal Brisbane and Women’s Hospital (RBWH), Brisbane, Australia. The RBWH is the largest hospital in Queensland and has nearly 1000 hospital beds. Patients with HF attending the between January 2018 to March 2018 were included in the study. Patients with documented evidence of HF were recruited to the study using convenient sampling method and upon recruitment, the diagnosis was confirmed by the clinical cardiology staff.

Following informed consent the study participants completed a three-sectioned questionnaire. The first section included the socio-demographic information such as age and sex and diagnosis of the patient. The second section included the five-level EQ-5D questionnaire (EQ-5D-5 L) and the third section included the Minnesota Living with Heart Failure questionnaire (MLHFQ). Institutional ethics committee approval was obtained from the Griffith University Human Research Ethics Committee (Reference no. 2017/069).

### Instruments

The source instrument for mapping was the MLHFQ and the target instrument was the EQ-5D-5 L.

### EuroQol five-dimensional questionnaire (EQ-5D)

The three-level version EQ-5D is the most widely used preference-based instrument [17]. In 2011 the new version of the instrument, the five-level EQ-5D (EQ-5D-5 L) was developed to improve the ability of the instrument to measure small changes in the health state, especially in patients with milder conditions [18]. EQ. 5D is an instrument which has been used to assess quality of life of heart failure patients [19]. Furthermore, EQ-5D-5 L is a valid instrument to be used in health research in Australia [20]. This instrument contains five domains: mobility, self-care, usual activities, pain/discomfort and anxiety/depression. Each domain has one item and each item has five response levels with one denoting no problems and five denoting extreme problems. Thus, EQ-5D-5 L can define mutually exclusive 3125 different health states [21]. Since then EQ-5D-5 L has been used to measure health state utility of different disease conditions [22–24] including cardiovascular disease [25, 26]. In this study the EQ-5D-5 L was scored using the widely used UK tariff [21] since by far the Australian-specific tariff is not yet available. Therefore, in Australia, UK tariffs are commonly used to calculate EQ-5D-5 L utility scores [27, 28]. The EQ-5D-5 L utility scores (based on the UK tariff) range from − 0.594 (the worst health state) to 1.0 (the best health state), whilst 0 equals being dead and negative values represent health status considered worse than “dead”.

### Minnesota living with heart failure questionnaire (MLHFQ)

The MLHFQ is a self-administered, 21-item disease-specific instrument for patients with heart failure [29]. MLHFQ is an instrument which has been widely used to assess quality of life among heart failure patients [30–32]. Each item is scored in a 6-point Likert Scale (0 to 5), thus the total score could range from 0 to 105, with higher scores indicating more significant impairment in health-related quality of life. The MLHFQ has two domains; physical domain (eight items, score range from 0 to 40) and emotional domain (five items, score range from 0 to 25).

### Statistical analysis

The patient characteristics were summarized using mean (standard deviation [SD]) and median for continuous variables while frequency (percentage) was used for the categorical variables. Normality of the continuous variables was assessed using the Shapiro-Wilks test. A scatter plot and Spearman correlation coefficient were used to describe the correlation between the MLHFQ score and the EQ-5D-5 L utility score. The magnitude of the correlation coefficients (r) were interpreted according to Guilford’s criteria [33]. According to this criteria the correlation coefficients are divided in to five categories depending on the strength of the association, namely; very low (r: 0.00–0.20), low (r: 0.21–0.40), moderate (r: 0.41–0.60), high (r: 0.61–0.80) and very high (r: 0.81–1.00).

Both direct and indirect response mapping were conducted in the study.

The best regression method to develop a predictive model is a widely discussed topic. The current consensus is that there is no one method that fits all data sets [34]. To get around this uncertainty, during direct mapping, six regression techniques were used on the same dataset and the best was chosen based on validation parameters. Six regression techniques, each of which has the capability to cope with either skewness, heteroscedasticity, ceiling effects and/or the potential presence of outliers in the data set [35], were used to identify the optimal mapping functions for each of the three models:

- *Ordinary least square (OLS)*

In the OLS the coefficients and the intercept are calculated by minimising the sum of the squares of the differences between the observed and predicted utility scores. The model assumes that the errors are normally distributed with mean zero and has a constant variance (homoscedasticity) [36]. The OLS is the most widely reported method in mapping literature although violating the above assumptions [35].

- *Generalized linear modelling (GLM)*

GLM allows the errors to have a skewed distribution by having a priori specifying the distribution. All potential combination of family and link functions were investigated and the ones with the best mapping performance was chosen for each model. For the Models 1 and 2, gamma distribution and identity link function produced the best prediction model, while Gaussian distribution and ‘identity’ link function (which is equivalent to the OLS) produced the best prediction model for the Model 3.

- *Censored least absolute deviations (CLAD)*

This method is best suited for outcome variables censored at lower or upper endpoints. This method uses median parameters rather than means, thus robust to distributional assumptions and heteroscedasticity [37].

- *Multivariable fractional polynomials (MFP)*

MFP is a useful modelling technique to be used when the dependant and the independent variables have a non-linear relationship [38]. Different regression methods were tested (such as OLS and GLM mentioned above) and the median regression produced the best prediction model in all three models.

- *Robust MM estimator (MM)*

This method is useful when presence of either heteroscedasticity or outliers limit the use of traditional regression methods [39].

- *Beta regression model (BETA)*

This method is robust to skewness and can estimate both unimodal and bimodal utilities [35].

The direct mapping algorithms were developed using aforementioned regression techniques. In particular, three model specifications were considered to predict the EQ-5D-5 L utility score mainly using MLHFQ total score (Model 1), MLHFQ domain scores (Model 2), or MLHFQ item scores (Model 3) (see below). Based on the previous literature [40, 41], squared terms of MLHFQ total score, MLHFQ domain scores and MLHFQ item scores were added as independent variables to the linear modes (i.e. OLS, CLAD, MM) in order to account for the non-linear relationship between EQ-5D-5 L utility values and MLHFQ. However, for the non-linear models, i.e. the GLM, MFP, and BETA, we only included the original term in the modelling since the potential nonlinear relationship will be considered during the modelling process. Socio-demographic characteristics such as age and sex were included in the models to improve the predictive performance. Forward stepwise regression method was used to identify the statistically significant predictors (i.e. *P* <  0.05) to be included in the final mapping functions.

$$ \mathrm{EQ}\ 5\mathrm{D}\ 5\;\mathrm{L}\ \mathrm{utility}\ \mathrm{score}={\upbeta}_0+{\upbeta}_1\ast \mathrm{MLHFQ}\ \mathrm{total}\ \mathrm{score}+{\upbeta}_2\ast \mathrm{MLHFQ}\ {\mathrm{total}\ \mathrm{score}}^2+{\upbeta}_3\ast \mathrm{Age}+{\upbeta}_4\ast \mathrm{Sex}\ \left(\mathrm{Model}\ 1\right) $$

$$ \mathrm{EQ}\ 5\mathrm{D}\ 5\;\mathrm{L}\ \mathrm{utility}\ \mathrm{score}={\upbeta}_0+{\upbeta}_1\ast \mathrm{MLHFQ}\ \mathrm{Physical}\ \mathrm{domain}\ \mathrm{score}+{\upbeta}_2\ast \mathrm{MLHFQ}\ \mathrm{Physical}\ {\mathrm{domain}\ \mathrm{score}}^2+{\upbeta}_3\ast \mathrm{MLHFQ}\ \mathrm{Emotional}\ \mathrm{domain}\ \mathrm{score}+{\upbeta}_4\ast \mathrm{MLHFQ}\ \mathrm{Emotional}\ {\mathrm{domain}\ \mathrm{score}}^2+{\upbeta}_5\ast \mathrm{Age}+{\upbeta}_6\ast \mathrm{Sex}\ \left(\mathrm{Model}\ 2\right) $$

$$ \mathrm{EQ}\ 5\mathrm{D}\ 5\;\mathrm{L}\ \mathrm{utility}\ \mathrm{score}={\upbeta}_0+\sum \limits_{j=1}^m{\beta}_j\ast \mathrm{MLHFQ}\ \mathrm{item}\ \mathrm{score}\mathrm{s}+\sum \limits_{j=1}^m{\beta}_j\ast \mathrm{MLHFQ}\ {\mathrm{item}\ \mathrm{score}\mathrm{s}}^2+{\upbeta}_2\ast \mathrm{Age}+{\upbeta}_3\ast \mathrm{Sex}\ \left(\mathrm{Model}\ 3\right) $$

In the indirect response mapping, EQ. 5D 5 L responses were predicted separately using the MLHFQ item scores using ordered logit model [42]. This will produce a set of mapping algorithms which will predict each of the 5 EQ. 5D 5 L dimension responses. This will enable calculating country-specific EQ-5D-5 L utilities by applying country-specific tariffs, not just the UK tariff that was used for this study. The MLHFQ items that should be used to predict each of the EQ. 5D 5 L dimension responses were selected using forward stepwise regression technique.

$$ \mathrm{EQ}\ 5\mathrm{D}\ 5\;\mathrm{L}\ \mathrm{response}\ \left(\mathrm{eg}.\mathrm{Mobility}\right)=\sum \limits_{j=1}^m{\beta}_j\ast \mathrm{MLHFQ}\ \mathrm{item}\ \mathrm{scores} $$

### Assessing model performance

Goodness-of-fit of the models were mainly assessed using mean absolute error (MAE) and the root mean square error (RMSE). MAE was computed as the mean of the absolute differences between the predicted and actual observed EQ-5D-5 L utilities, while the root square value of the mean squared differences between the actual and predicted EQ-5D-5 L utilities was considered as RMSE. However, more weight was given to MAE as it is easily interpretable and considered to be less sensitive to outliers [43].

Furthermore, four additional criteria were also considered to assess the models

- exactness of the predicted sample mean
- the range of predictions
- the proportion of predicted utilities deviating from observed values by absolute error <  0.03 and <  0.05
- intra-class correlation coefficients

In the absence of an external validation dataset, predictive performance of the models were assessed using 3-fold cross validation method [44, 45]. The data set was randomly divided in to three equal-sized sections using random number generation algorithms. During each iteration, two groups (67% of the data set) were allocated to the “estimation sample” and all six regression models were applied to develop the coefficients. Then the remaining group (33% of the data set) was used as the ‘validation sample’, where the estimates generated during the previous step were used to estimate the predicted values for the ‘validation sample’. This process was repeated three times, so as to make certain that each of the three subgroups was used in the estimation and validation iterations. Thereafter, the validation results were pooled together and model performance based on the pooled estimated goodness-of-fit statistics (MSE and MAE) was assessed.

The “Mapping onto Preference-based measures reporting Standards” (MAPS) checklist was followed in this study [46]. All statistical analyses were conducted using STATA Software version 15.0.

## Results

### Sample characteristics

**Fig. 1:**
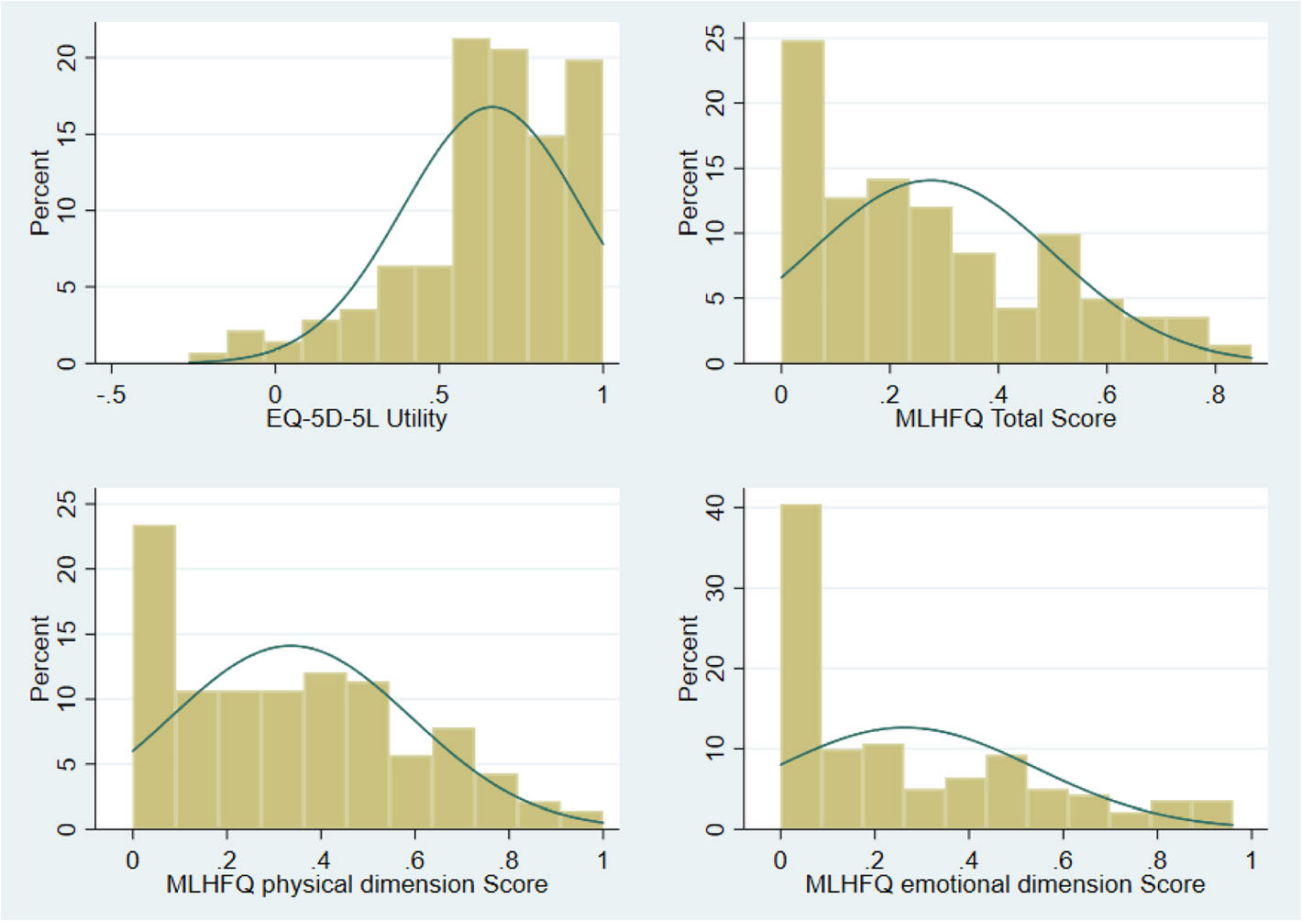
Histogram of the MLHFQ total and domain scores and EQ-5D-5 L

**Fig. 2:**
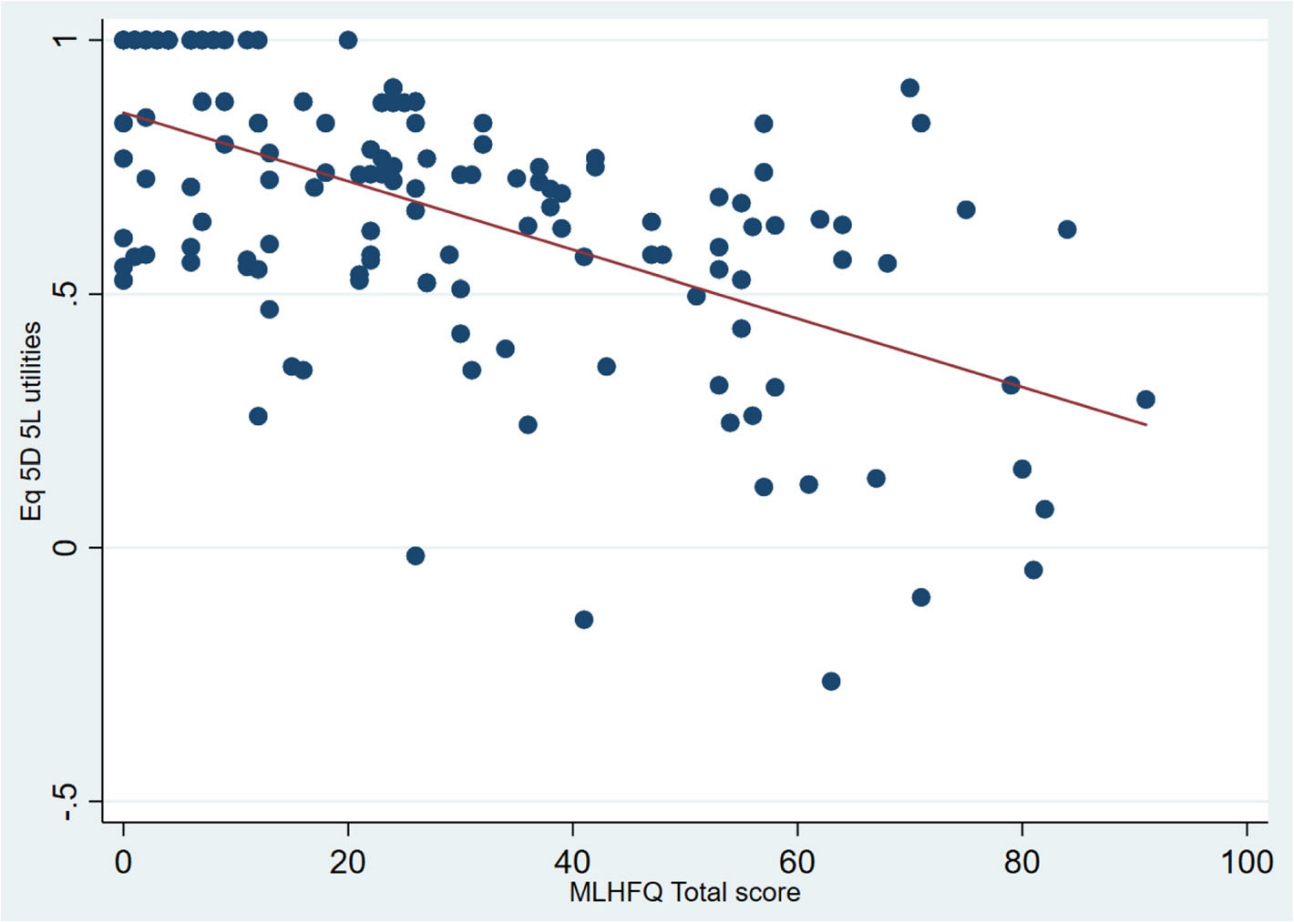
Scatter plot between the MLHFQ total score and the EQ-5D-5 L utilities

A total of 141 patients diagnosed with heart failure participated in the study. The mean age of the study participants was 63.3 (SD 14.8) years and more than half (*n* = 96; 68.0%) of them were males (Table 1). The mean and the median EQ-5D-5 L utility scores were 0.6619 (SD 0.27) and 0.708 (0.553–0.877) respectively. The mean MLHFQ total score was 28.9 (SD 23.5). Frequency distribution plots of EQ-5D-5 L and MLHFQ total score is depicted in Fig. 1. EQ-5D-5 L utility values were negatively skewed while the MLHFQ total score was positively skewed, indicating that both values were non-normally distributed. This was further proven by Shapiro Wilks test of normality (*p* <  0.001). A moderately strong negative correlation was observed between EQ-5D-5 L utility scores and MLHFQ total score (Spearman correlation coefficient (r) = − 0.580; *p* <  0.001) (Fig. 2). Similarly, MLHFQ physical domain score (Spearman correlation coefficient (r) = − 0.5773; *p* <  0.001) and MLHFQ emotional domain score (Spearman correlation coefficient (r) = − 0.5498; *p* < 0.001) were also moderately correlated with the EQ-5D-5 L utility score.

**Table 1:** Participants characteristics (N = 141)

Characteristic	
Age (Years)	
Mean (SD)	63.3 (14.8)
Median (IQR)	66.0 (54.0–74.0)
Sex	
Male (%)	96 (68.0)
Female (%)	45 (32.0)
EQ-5D-5 L utility score	
Mean (SD)	0.6619 (0.27)
Median (IQR)	0.708 (0.553–0.877)
MLHFQ total score (Max score 105)	
Mean (SD)	28.9 (23.5)
Median (IQR)	24.0 (09.0–27.0)
MLHFQ physical domain score (Max score 40)	
Mean (SD)	13.4 (10.3)
Median (IQR)	13.0 (4.0–12.0)
MLHFQ emotional domain score (Max 25)	
Mean (SD)	6.6 (6.9)
Median (IQR)	4.0 (0.0–12.0)

*MLHFQ* Minnesota Living with Heart Failure Questionnaire.

### Prediction of EQ. 5D 5 L utility scores

In the direct mapping, six regression methods and three model specifications were assessed separately. Age (*p* > 0.2) and sex (*p* > 0.8) were consistently insignificant in all regressions, thus they were excluded from the regression models. Of the 21 items in the instrument, only three items (item 04, 17 and 21) were found to be statistically significant in the forward stepwise regression method. Thus, only those three items were included in the final equation of the Model 3. Furthermore, squared terms used in the OLS, CLAD, MM models were found to be not significant, thus were removed from the final model.

Similar to direct mapping, age (*p* > 0.05) and sex (*p* > 0.05) were not significant in the indirect mapping response as well. Of the 21 items in the instrument, the items which were statistically significant in predicting each of the EQ-5D-5 L dimension responses (selected using the forward stepwise regression method) are indicated below.

Mobility ➔ Item 3, Item 5 and Item 15.

Self-care ➔ Item 4, Item 15, Item 16 and Item 17.

Usual activity ➔ Item 2, Item 4, Item 14, Item15 and Item 17.

Pain/Discomfort ➔ Item 1 and Item 13.

Anxiety/depression ➔ Item 2, Item 4 and Item 21.

Table 2 summarised the key goodness-of-fit statistics for different model and method combinations based on the full sample (both direct and indirect response mapping). In the direct mapping, all models under predicted the maximum observed utility value (1.000), while all models over predicted the lowest observed utility (− 0.2630). The highest discrepancies between the predicted minimum and the observed minimum was found in MM estimates (Model 1) and lowest was in Beta (Model 3). Regarding the RMSE and MAE values, OLS and GLM had the lowest RMSE value in each of the three model specifications. However, the lowest MAE was recorded from the MFP model in all three-model specifications.

**Table 2:** Goodness of fit results from full estimation sample (N = 141)

Model Specification	Mean utility (SD)	Minimum	Maximum	P.25	Median	P.75	RMSE	MAE	Abs diff < 0.03	Abs diff < 0.05	ICC
**Observed**	0.6619	−0.2630	1.0000	0.5530	0.7080	0.8770					
**Model 1**											
OLS	0.6619	0.2421	0.8575	0.5396	0.6952	0.7966	0.2219	0.1782	9.9	16.2	0.670
GLM	0.6619	0.2421	0.8575	0.5396	0.6952	0.7966	0.2219	0.1782	9.9	16.2	0.670
CLAD	0.6849	0.2920	0.8679	0.5705	0.7160	0.8109	0.2234	0.1740	11.3	18.3	0.654
MFP	0.7164	0.2679	0.9254	0.5858	0.7520	0.8604	0.2288	**0.1713**	12.7	18.3	0.684
MM	0.7365	0.3104	0.9350	0.6124	0.7703	0.8732	0.2342	0.1724	12.7	19.7	0.673
BETA	0.7267	0.1593	0.9253	0.5885	0.8044	0.8912	0.2386	0.1778	10.6	16.2	0.705
**Model 2**											
OLS	0.6619	0.2308	0.8631	0.5519	0.6818	0.8028	0.2185	0.1750	9.2	15.5	0.690
GLM	0.6619	0.2308	0.8631	0.5519	0.6818	0.8028	0.2185	0.1750	9.2	15.5	0.690
CLAD	0.7066	0.2762	0.9072	0.5964	0.7272	0.8473	0.2230	0.1689	10.6	19.0	0.689
MFP	0.7224	0.2920	0.9263	0.6132	0.7414	0.8653	0.2268	**0.1684**	11.3	16.9	0.688
MM	0.7373	0.2897	0.9429	0.6125	0.7586	0.8871	0.2313	0.1695	9.2	16.2	0.697
BETA	0.7256	0.1505	0.9335	0.6154	0.7887	0.9019	0.2349	0.1761	4.2	12.0	0.717
**Model 3**											
OLS	0.6619	0.2360	0.8222	0.5697	0.6984	0.8222	0.2151	0.1721	11.3	16.9	0.707
GLM	0.6619	0.2360	0.8222	0.5697	0.6984	0.8222	0.2151	0.1721	11.3	16.9	0.707
CLAD	0.6967	0.2920	0.8370	0.6126	0.7322	0.8370	0.2217	0.1703	13.4	22.5	0.667
MFP	0.7086	0.2883	0.8655	0.6177	0.7473	0.8655	0.2204	**0.1676**	13.4	20.4	0.700
MM	0.7260	0.2719	0.8953	0.6278	0.7795	0.8953	0.2248	0.1695	9.9	16.9	0.718
BETA	0.7345	0.1775	0.9032	0.6098	0.8027	0.9032	0.2321	0.1745	10.6	16.9	0.731
**Indirect mapping**											
OLOGIT	0.6443	0.4790	0.7680	0.5410	0.5920	0.7680	0.2363	0.1927	10.6	15.5	0.512

Dependant variable: EQ-5D-5 L utility score; Independent variables: Model 1 - MLHF total score; Model 2 – MLHF domain scores; Model 3 – MLHF item scores (Item 04, 17 and 21).

Abs diff. < 0.03 (0.05)% - proportion of predicted utilities whose absolute values deviate from the mean of the observed utility values by less than 0.03 (0.05); P.25 – 25th percentile; *P.75* 75th percentile, *RMSE* Root Mean Square Error; *MAE* Mean Absolute Error; ICC – Intra Class Correlation.

*OLS* Ordinary least square, *GLM* Generalized linear modelling, *CLAD* Censored least absolute deviations, *MFP* Multivariable fractional polynomials, *MM* Robust MM estimator, *BETA* Mixture beta regression model, OLOGIT ordered logit (indirect response mapping).

Regarding the indirect response mapping, results show that the performance was comparable with the direct mapping approach based on RMSE but was worse based on MAE.

### Validation

In the absence of an external validation dataset, predictive performance of the models was assessed using three-fold cross validation method (Table 3). All models were assessed for goodness of fit using the MAE and RMSE and a consistent pattern was seen in all three-model specifications (direct mapping); OLS and GLM showed the lowest RMSE value and MFP estimates showed the lowest MAE value. Based on the results in Table 3, it is concluded that mapping algorithms developed using MFP regression technique exhibited the best predictive ability to predict the EQ-5D-5 L utility score using MLHFQ total score, MLHFQ domain scores and MLHFQ item scores.

**Table 3:** Goodness of fit results from validation analysis

	Validation method (3-fold) Pooled sample (***N*** = 141)				
	Mean utility	RMSE	MAE	Ab diff < 0.03	Ab diff < 0.05
**Observed**	0.6619				
**Model 1**					
OLS	0.6624	0.2220	0.1783	9.9	16.2
GLM	0.6153	0.2220	0.1916	3.5	9.2
CLAD	0.6925	0.2240	0.1730	12.7	19.7
MFP	0.7164	0.2288	0.1713	12.7	18.3
MM	0.7254	0.2309	0.1717	12.0	20.4
BETA	0.7246	0.2378	0.1775	10.6	16.2
**Model 2**					
OLS	0.6696	0.2187	0.1738	8.5	16.2
GLM	0.6711	0.2197	0.1740	9.2	15.5
CLAD	0.7304	0.2316	0.1710	7.0	21.8
MFP	0.7224	0.2268	0.1684	11.3	16.9
MM	0.7313	0.2292	0.1688	11.3	16.9
BETA	0.7286	0.2355	0.1761	4.9	12.0
**Model 3**					
OLS	0.6671	0.2153	0.1711	12.0	17.6
GLM	0.6671	0.2153	0.1711	12.0	17.6
CLAD	0.7173	0.2268	0.1700	11.3	14.8
MFP	0.7086	0.2204	0.1676	13.4	20.4
MM	0.7177	0.2229	0.1689	9.2	16.9
BETA	0.7347	0.2318	0.1740	11.3	16.9
**Indirect mapping**					
OLOGIT	0.6498	0.2353	0.1935	8.5	12.7

Dependant variable: EQ-5D-5 L utility score; Independent variables: Model 1 - MLHF total score; Model 2 – MLHF domain scores; Model 3 – MLHF item scores (Item 04, 17 and 21).

Abs diff. < 0.03 (0.05)% - proportion of predicted utilities whose absolute values deviate from the mean of the observed utility values by less than 0.03 (0.05); RMSE – Root Mean Square Error; MAE – Mean Absolute Error.

OLS - Ordinary least square; GLM - Generalized linear modelling; CLAD - Censored least absolute deviations; MFP - Multivariable fractional polynomials; MM - Robust MM estimator; BETA - Mixture beta regression model; OLOGIT ordered logit (indirect response mapping).

Regarding the indirect response mapping, validation results was very much similar to the results of the main sample.

### Best performing models

**Fig. 3:**
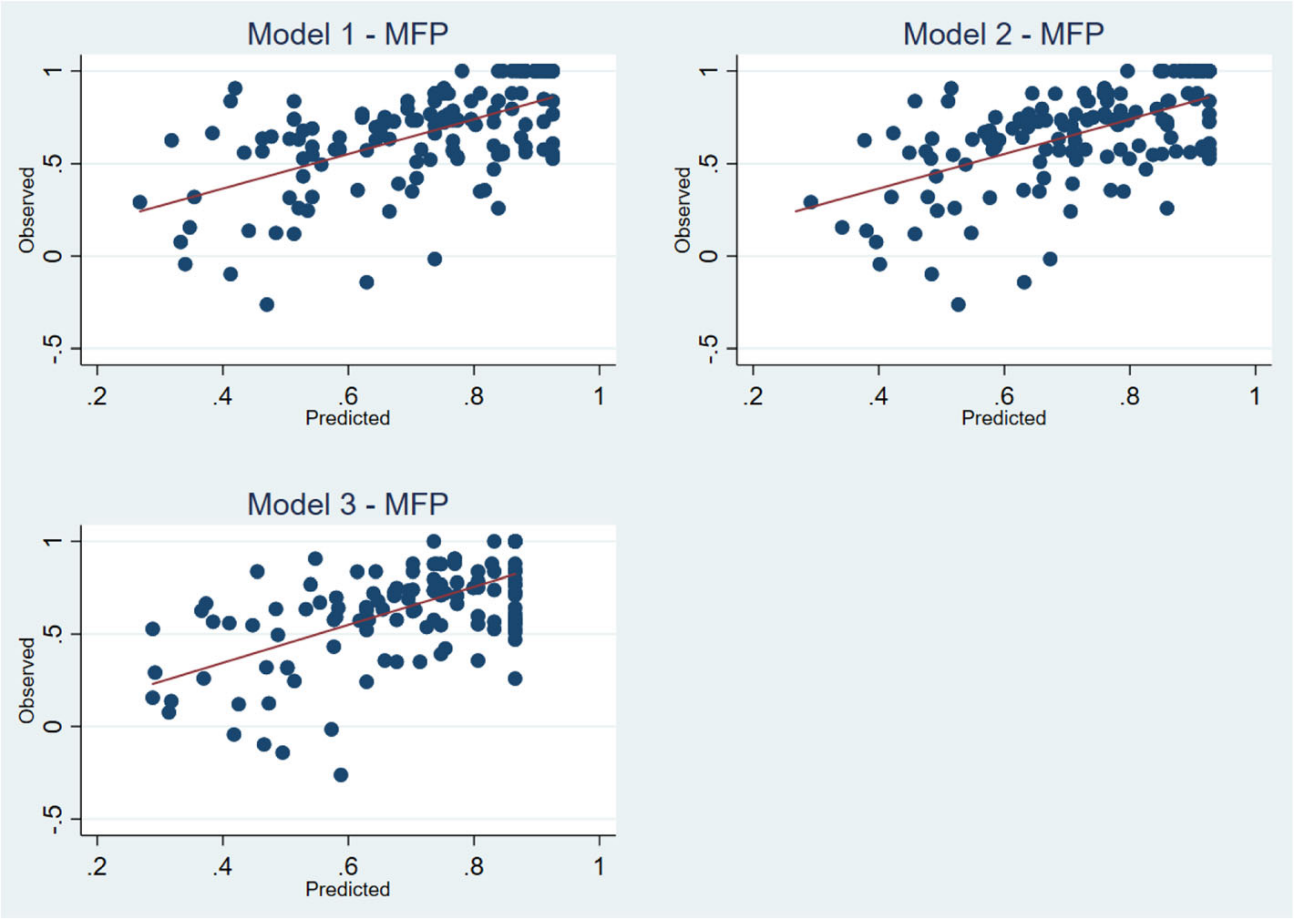
Scatter plot of observed versus predicted EQ-5D-5 L. Line of equality between observed and predicted values (red line)

**Fig. 4:**
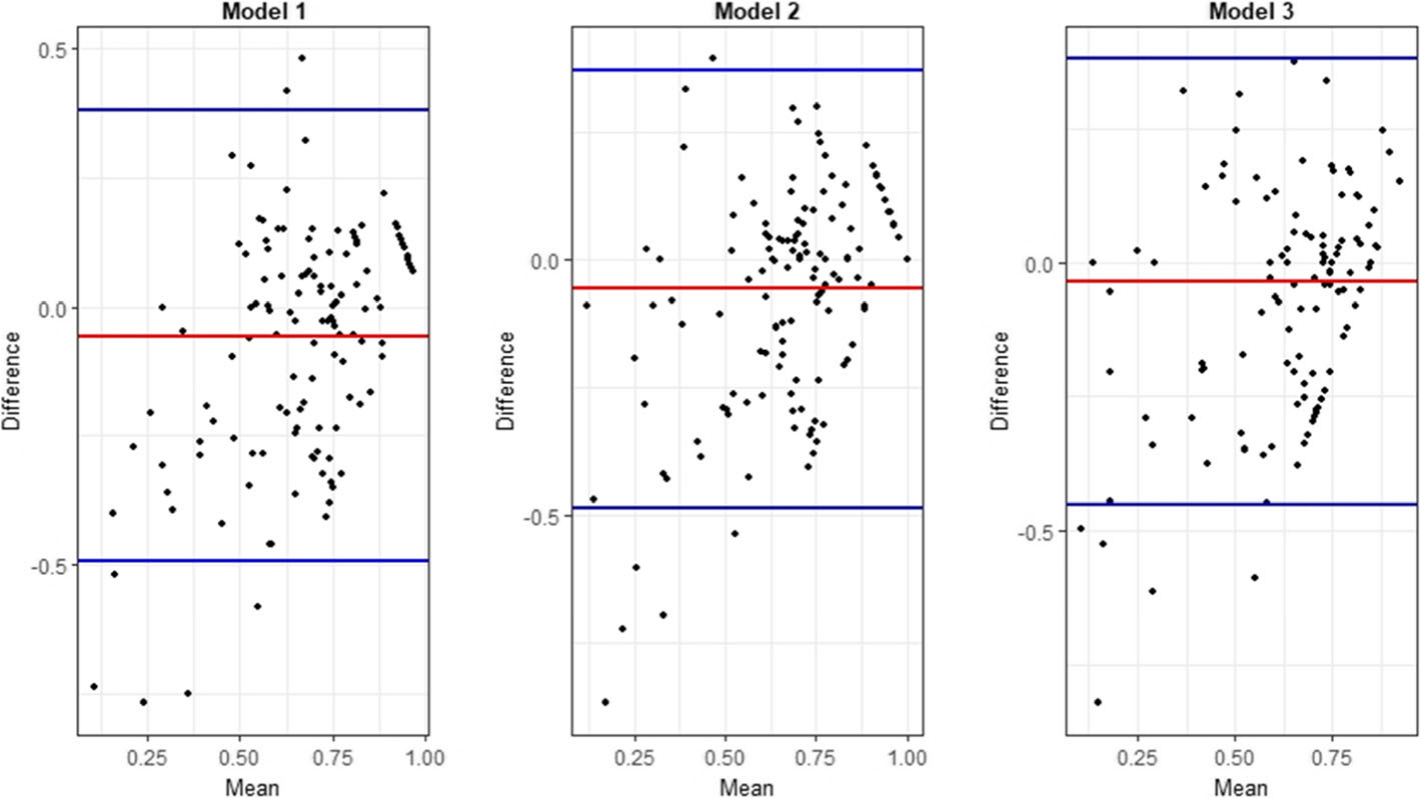
Bland and Altman plot of differences between the actual and the predicted EQ-5D-5 L utility scores

The best models to predict the EQ-5D-5 L utility score using MLHFQ total score, MLHFQ domain scores and MLHFQ item scores were selected on the basis of their performance in the cross validation step, with more weight put on the MAE following evidence in the literature [43]. MFP regression technique performed best in all three-model specifications. Detailed Goodness-of-fit indicators for the above three models are indicated in the Table 4. Fig. 3 illustrates the scatter plots of observed vs predicted EQ-5D-5 L using the selected best performing models. Table 4 and Fig. 3 indicate that the model over predicted the severe health states. For example, in Model 1, observed 50th and 75th percentile were 0.7520 and 0.8604 while the predicted values were 0.7080 and 0.8770. However, in the Model 1 the observed 5th and 10th percentile were 0.0826 and 0.2593 while the predicted values were 0.3653 and 0.4313. The Bland-Altman plot showed proportional error and wide limits of agreement (Fig. 4).

**Table 4:** Model performance for the best fitting model (Direct mapping)

Model Specification	Mean utility (SD)	Min	P.25	Median	P.75	Max	RMSE	MAE	Abs diff < 0.03	Abs diff < 0.05	ICC
**Observed**	0.6619	−0.2630	0.5530	0.7080	0.8770	1.0000					
**Model 1 (MFP)**											
Cross validation sample	0.7164	0.2679	0.5858	0.7520	0.8604	0.9254	0.2288	0.1713	12.7	18.3	0.513
**Model 2 (MFP)**											
Cross validation sample	0.7224	0.2920	0.6132	0.7414	0.8653	0.9263	0.2268	0.1684	11.3	16.9	0.520
**Model 3 (MFP)**											
Cross validation sample	0.7086	0.2883	0.6177	0.7473	0.8655	0.8655	0.2204	0.1676	13.4	20.4	0.517

Dependant variable: EQ-5D-5 L utility score; Independent variables: Model 1 - MLHF total score; Model 2 – MLHF domain scores; Model 3 – MLHF item scores.

Abs diff. < 0.03 (0.05)% - proportion of predicted utilities whose absolute values deviate from the mean of the observed utility values by less than 0.03 (0.05); P.25 – 25th percentile; P.75 – 75th percentile; RMSE – Root Mean Square Error; MAE – Mean Absolute Error; ICC – Intra Class Correlation.

MFP Multivariable fractional polynomials.

Table 5 reports the detailed MFP regression coefficients for each model specification, which can be used to predict the EQ-5D-5 L utility score in the three specification scenarios. The transformation scores of the MFP models are as follows.

**Table 5:** Direct mapping equation from MLHFQ to EQ. 5D 5 L utility scores

	Model 1	Model 2	Model 3
	MFP coefficient (SE)	MFP coefficient (SE)	MFP coefficient (SE)
MLHF score	−0.0072258 (0.0011835)		
MLHF Physical domain		−0.011195 (0.0040171)	
MLHF Emotional domain		−0.0081086 (0.0059928)	
MLHF Item 04 score			−0.0591304 (0.022508)
MLHF Item 17 score			−0.0370435 (0.0227411)
MLHF Item 21 score			−0.0333478 (0.0207565)
Constant	0.7164347 (0.0276502)	0.7223581 (0.0253515)	0.7085862 (0.0251183)

Model 1 - MLHF total score; Model 2 – MLHF domain scores; Model 3 – MLHF item scores; MFP - Multivariable fractional polynomials

All coefficients reported in Table 5 were statistically significant (all *P* < 0.05)

MLHF transformation factor - 28.92198582.

MLHF Physical domain transformation factor - 13.43971631.

MLHF Emotional domain transformation factor - 6.595744681.

MLHF Item 04 score transformation factor - 1.304964539.

MLHF Item 17 score transformation factor - 1.042553191.

MLHF Item 21 score transformation factor - 1.234042553.

For example, the EQ-5D-5 L utility score could be predicted from MLHFQ total score using the following equation.

#### MFP

Step 1 – calculate a transformed MLHF total score.

MLHF transformed total score* = MLHF total score - 28.*92198582.*

Step 2 – calculate the predicted EQ-5D-5 L utility score.

EQ-5D-5 L utility predicted using MFP = 0.7164347–0.0072258 x MLHF transformed total score*.

Calculating the country specific utility values using indirect response mapping algorithms is a three-step process.

**Step 1:** Calculate the response score using the relevant coefficients (Table 6).

**Table 6:** Indirect response mapping equations from MLHF item scores to each EQ-5D-5 L dimension (order logit estimates), *N* = 141

	MO	SC	UA	PD	AD
Item 1				0.3193945 (0.1152699^*^	
Item 2			0.3698597 (0.1398624) ^*^		0.4219077 (0.1323218)^*^
Item 3	0.2759361 (0.126163) ^*^				
Item 4		0.6020205 (0.1597379)^**^	0.3992772 (0.1709487) ^*^		−0.3880844 (0.1626194)^*^
Item 5	0.5664895 (0.1299027) ^**^				
Item 13				0.4858658 (0.1064107^**^	
Item 14			−0.3815404 (0.1724133) ^*^		
Item 15	0.2733695 (0.1090548) ^*^	0.3565042 (0.1409699)^*^	0.2886012 (0.1204365)^*^		
Item 16		−0.465391 (0.1704747)^*^			
Item 17		0.3629092 (0.1556987)^*^	0.4555838 (0.1507831)^*^		
Item 21					0.7580628 (0.1365814)^**^
/ Cut 1	1.102055 (0.2983015)	1.941272 (0.319719)	1.268015 (0.3006514)	0.6358669 (0.293458)	0.8957798 (0.2790692)
/ Cut 2	2.64014 (0.367798)	3.927365 (0.4843209)	3.28451 (0.4111717)	2.320936 (0.3529089)	2.760695 (0.3730153)
/ Cut 3	4.632778 (0.5054664)	4.942844 (0.5975513)	5.099221 (0.5510285)	4.237298 (0.4816227)	4.300851 (0.4881258)
/ Cut 4	6.351936 (0.7130962)	5.918955 (0.7567116)	6.257307 (0.6722751)	6.962672 (01.088636)	5.742625 (0.6660593)

EQ-5D dimensions: MO mobility, SC self-care, UA usual activities, PD pain/discomfort, AD anxiety/depression, /cut# estimated cut points; **p < 0.001, *p < 0.05. Standard errors in parentheses.

E.g.; Calculating EQ-5D-5 L mobility response score.

MLHF items needed: item 3, item 5 and item 15 (see Table 6).

MLHF scores of each item (example): 5 (item 3), 3 (item 5) and 3 (item 15).

Predicted EQ-5D-5 L mobility response score = ∑ (Item score x Coefficient).

= (5 × 0.275936) + (3 × 0.56649) + (3 × 0.27337).

= 3.899258.

**Step 2**: Check the predicted EQ-5D-5 L mobility response score against the cut-off values (see Table 6).

Predicted EQ-5D-5 L mobility response score ➔ 3.899258.

Cut-offs in Mobility response.

Less than 1.102055 ➔ Response 1.

Between 1.102055 and 2.64014 ➔ Response 2.

Between 2.64014 and 4.632778 ➔ Response 3.

Between 4.632778 and 6.351936 ➔ Response 4.

More than 6.351936 ➔ Response 5.

Since 3.899258 is between 2.64014 and 4.632778 the final EQ-5D-5 L mobility response is 3. Conduct the same process for all other EQ-5D-5 L domains.

**Step 3**: Appy country-specific tariffs to calculate the country-specific EQ-5D-5 L utilities.

## Discussion

This is the first study to map MLHF onto EQ-5D-5 L utility scores using both direct and indirect response mapping techniques. Any previous study which did not use a preference-based instrument, but included MLHF questionnaire for data collection can use this algorithm to calculate utility values and use them in estimating cost effectiveness of the intervention in cost per QALY terms. Our regression analyses showed that the EQ-5D-5 L utility scores of heart failure patients in our sample was best predicted by the MFP regression model. Furthermore, results indicated that the indirect response mapping algorithms can be used effectively to calculate country specific utility values.

The mean MLHFQ score in the study population was 28.7 (SD 23.5). However, mean MLHFQ values available in the literature show a wide variation. According to Fu et al. (2016) [47] and Mogle et al. (2017) [48] who have validated the MLHFQ to Taiwan and Spanish populations, the mean MLHFQ score was 25.3 and 27.8 respectively. But a couple of studies have reported higher mean MLHFQ scores [49, 50]. Therefore, the sample used in the present study may not represent the wide spectrum of HF population. Concurrent validity between two instruments implies the conceptual overlap between the two instruments, and evidence indicate that this is an important determinant of a successful mapping analysis [51, 52]. In the present study a moderately strong negative correlation was observed between EQ-5D-5 L utility scores and MLHFQ total score (r = − 0.580), MLHFQ physical domain score (r = − 0.5773) and MLHFQ emotional domain score (r = − 0.5498), implying good concurrent validity.

The best models to predict the EQ-5D-5 L utility score were selected on the basis of their performance in the cross validation step, with more weight put on the MAE following evidence in the literature [43]. The choice of an error measure can affect selection of the best performing model. RMSE is strongly influenced by scale and more sensitive to outliers. However, MAE is easily interpretable, avoids the need for trimming and considered to be less sensitive to outliers in [43]. Therefore, MAE is considered a reliable error measure. MFP regression technique performed best in all three-model specifications, i.e. had the lowest MAE.

Absence of previous comparable mapping studies between MLHFQ and EQ-5D-5 L, precludes direct comparison of validity parameters of this study with literature. However, in the present study the validity criteria used to evaluate the regression models to select the best predictive model, indicated mixed results. The MAE value of all three models were relatively higher compared to the values reported in the literature [13, 40]. Furthermore, the RMSE values were also relatively higher in the present study indicating that the absolute deviation from the observed values is higher. In our analysis all three models over predicted the more severe health states. The minimum observed value was − 0.2630, but the predicted minimum values in the three models were 0.1593, 0.1505 and 0.1775. This narrow range of the predicted values, which is commonly reported in the mapping literature [53, 54], could explain the relative high RMSE values in the present study. Association of substantial decrements in utility weights of EQ-5D-5 L in the severe health states and conceptual differences between the two instruments are believed to be the reasons for this commonly observed narrow range of the predicted values [55, 56]. Thus, the algorithms presented in this paper should only be used to predict the mean utility score of a sample, but should not be used to make individual predictions.

We also conducted indirect response mapping to predict the responses to each of the EQ-5D-5 L dimensions. This will enable calculation of different utility values from different country-specific value sets of the EQ-5D-5 L, thus the reported indirect mapping algorithms can be used by researches of other countries as well. However, it is important to note that the results of the indirect mapping algorithms depend on whether the heart failure patients in other countries will have a similar response pattern to the patients reported in the present study.

This study has several strengths. Firstly, we used six regression methods to predict EQ-5D-5 L to account for the distribution of the data in the sample. The OLS is less suited for data sets with skewed distributions and homoscedasticity [36]. The GLM allows the errors to have a skewed distribution by having a priori specifying the distribution. MM-estimator is useful in the presence of either heteroscedasticity or outliers [39]. Beta regression is robust to skewness [35]. However, despite the strong assumptions, MFP performed superior to all the other regression methods used. The MFP is a useful modelling technique to be used when the dependant and the independent variables have a non-linear relationship [38]. Superiority of MFP compared to other robust regression models has been demonstrated previously as well [57]. Secondly, this is the only available algorithm in the medical literature which convers HF specific quality of life scores in to EQ-5D-5 L utility scores.

This study is not without limitations. Our sample size was 142. Although mapping studies have been conducted with similar sample sizes [51, 54] it is recommended to conduct further mapping studies using a larger sample size to evaluate the reliability of the mapping algorithm reported in the present study. Secondly, this model was validated using an internal sample, however validation using an external sample would have been ideal. Thirdly, since the presented algorithm may *over*-estimate the severe health states, they may *under*-estimate the utility gain in a study. Fourthly, in the present study the EQ-5D-5 L was scored using the UK tariff since the Australian-specific tariff is not available at present.

## Conclusion

In conclusion, to the authors’ knowledge, this is the first algorithm in the literature which converts HF specific quality of life scores onto EQ-5D-5 L utility scores using both direct and indirect response mapping techniques. The reported mapping algorithms would facilitate calculation of QALY in CUA related to heart failure.

## Data Availability

The datasets used and/or analysed during the current study are available from the corresponding author on reasonable request.

## References

[1] Dutka M, Bobinski R, Korbecki J. The relevance of microRNA in post-infarction left ventricular remodelling and heart failure. Heart failure reviews. 2019. 10.1007/s10741-019-09770-9 PMC6560007 30710255

[2] Ponikowski P, Anker SD, AlHabib KF, Cowie MR, Force TL, Hu S (2014). Heart failure: preventing disease and death worldwide. ESC Heart Failure..

[3] Baik D, Reading M, Jia H, Grossman LV, Masterson CR. Measuring health status and symptom burden using a web-based mHealth application in patients with heart failure. Eur J Cardiovasc Nurs. 2019;1474515119825704. 10.1177/1474515119825704 PMC6433527 30681003

[4] Jurgens CY, Fain JA, Riegel B (2006). Psychometric testing of the heart failure somatic awareness scale. J Cardiovasc Nurs.

[5] Australian Institute of Health and Welfare. Cardiovascular disease: Australian facts 2011: Australian Institute of Health and Welfare; 2011 [Available from: https://www.aihw.gov.au/reports/heart-stroke-vascular-disease/cardiovascular-disease-australian-facts-2011/contents/table-of-contents.

[6] Australian Bureau of Statistics. Causes of Death 2017. 2018 [Available from: https://www.abs.gov.au/ausstats/abs@.nsf/mf/3303.0.

[7] Cowie MR, Anker SD, Cleland JG, Felker GM, Filippatos G, Jaarsma T (2014). Improving care for patients with acute heart failure: before, during and after hospitalization. ESC Heart Failure..

[8] Mathers C, Penm R. Health system costs of cardiovascular diseases and diabetes in Australia 1993–94: Australian Institute of Health and Welfare; 1999.

[9] Kularatna S, Whitty JA, Johnson NW, Scuffham PA (2013). Health state valuation in low-and middle-income countries: a systematic review of the literature. Value Health..

[10] Whitehead SJ, Ali S (2010). Health outcomes in economic evaluation: the QALY and utilities. Br Med Bull.

[11] Heniford BT, Walters AL, Lincourt AE, Novitsky YW, Hope WW, Kercher KW (2008). Comparison of generic versus specific quality-of-life scales for mesh hernia repairs. J Am Coll Surg.

[12] Malý M, Vondra V (2006). Generic versus disease-specific instruments in quality-of-life assessment of chronic obstructive pulmonary disease. Methods Inform Med.

[13] Chen G, Garcia-Gordillo MA, Collado-Mateo D, del Pozo-Cruz B, Adsuar JC, Cordero-Ferrera JM, et al. Converting Parkinson-Specific Scores into Health State Utilities to Assess Cost-Utility Analysis. Patient-Centered Outcomes Res. 2018:1–11. 10.1007/s40271-018-0317-5 29876865

[14] Cichosz SL, Ehlers LH, Hejlesen O (2016). Health effectiveness and cost-effectiveness of telehealthcare for heart failure: study protocol for a randomized controlled trial. Trials..

[15] Chen G, McKie J, Khan MA, Richardson JR (2015). Deriving health utilities from the macnew heart disease quality of life questionnaire. Eur J Cardiovasc Nurs.

[16] Wijeysundera HC, Tomlinson G, Norris CM, Ghali WA, Ko DT, Krahn MD (2011). Predicting EQ-5D utility scores from the Seattle Angina Questionnaire in coronary artery disease: a mapping algorithm using a Bayesian framework. Med Decision Making.

[17] Chen G, Khan MA, Iezzi A, Ratcliffe J, Richardson J (2016). Mapping between 6 multiattribute utility instruments. Med Decision Making..

[18] Herdman M, Gudex C, Lloyd A, Janssen M, Kind P, Parkin D (2011). Development and preliminary testing of the new five-level version of EQ-5D (EQ-5D-5 L). Qual Life Res.

[19] Stehlik J, Mountis M, Haas D, Palardy M, Ambardekar AV, Estep JD, Ewald G, Russell SD, Robinson S, Jorde U, Taddei-Peters WC. Quality of life and treatment preference for ventricular assist device therapy in advanced heart failure: A report from the REVIVAL study. J Heart Lung Transplant. 2020;39(1):27–36. 10.1016/j.healun.2019.11.006 PMC6942204 31822442

[20] Keeley T, Al-Janabi H, Lorgelly P, Coast J (2013). A qualitative assessment of the content validity of the ICECAP-A and EQ-5D-5 L and their appropriateness for use in health research. PloS One..

[21] Devlin NJ, Shah KK, Feng Y, Mulhern B, van Hout B (2018). Valuing health-related quality of life: An EQ-5 D-5 L value set for E ngland. Health Econ.

[22] Tamura K, Sato T, Noto S, Imamura T (2019). Health-related Quality of Life in Patients with Mild-to-moderate Alzheimer’s Disease: A Study Using the Five-level Version of European Quality of Life-5 Dimensions (EQ-5D-5 L). Brain nerve.

[23] Chimatiro GL, Rhoda AJ, De Wit L (2018). Stroke patients’ outcomes and satisfaction with care at discharge from four referral hospitals in Malawi: A cross-sectional descriptive study in limited resource. Malawi Med J.

[24] Ramirez-Moreno JM, Munoz-Vega P, Alberca SB, Peral-Pacheco D (2019). Health-Related Quality of Life and Fatigue After Transient Ischemic Attack and Minor Stroke. J Stroke Cerebrovasc Dis.

[25] Gao L, Moodie M, Chen G. Measuring subjective wellbeing in patients with heart disease: relationship and comparison between health-related quality of life instruments. Quality of life research: an international journal of quality of life aspects of treatment, care and rehabilitation. 2019. 10.1007/s11136-018-2094-y 30604342

[26] Hong SH, Lee JY, Park SK, Nam JH, Song HJ, Park SY (2018). The Utility of 5 Hypothetical Health States in Heart Failure Using Time Trade-Off (TTO) and EQ-5D-5 L in Korea. Clin Drug Invest.

[27] Cheng Q, Kularatna S, Lee XJ, Graves N, Pacella RE (2019). Comparison of EQ-5D-5 L and SPVU-5D for measuring quality of life in patients with venous leg ulcers in an Australian setting. Qual Life Res.

[28] McCaffrey N, Kaambwa B, Currow DC, Ratcliffe J (2016). Health-related quality of life measured using the EQ-5D–5 L: South Australian population norms. Health Qual Life Outcomes..

[29] Middel B, Bouma J, de Jongste M, van Sonderen E, Niemeijer MG, Crijns H (2001). Psychometric properties of the Minnesota Living with Heart Failure Questionnaire (MLHF-Q). Clin Rehabil.

[30] Bundgaard JS, Thune JJ, Gislason G, Fosbol EL, Torp-Pedersen C, Aagaard D, et al. Quality of life and the associated risk of all-cause mortality in nonischemic heart failure. International journal of cardiology. 2020. 10.1016/j.ijcard.2020.02.008 32046910

[31] Wilkening GL, Brune S, Saenz PF, Vega LM, Kalich BA (2020). Correlation between medication regimen complexity and quality of life in patients with heart failure.

[32] Uy V, Hays RD, Xu JJ, Fayers PM, Auerbach AD, Black JT, et al. Do the unlabeled response categories of the Minnesota Living with Heart Failure Questionnaire satisfy the monotonicity assumption of simple-summated scoring? Quality of life research: an international journal of quality of life aspects of treatment, care and rehabilitation. 2020. 10.1007/s11136-020-02422-8 PMC7195256 31993916

[33] Pretorius T (2004). Numbers, hypotheses and conclusions: A course in statistics for the social sciences, edited by C. Tredoux and K. Durrheim: book review. S Afr J Psychol.

[34] Lorena AC, Garcia LP, Lehmann J, Souto MC, Ho TK. How Complex is your classification problem? A survey on measuring classification complexity. ACM Computing Surveys (CSUR). 2019;52(5):1–34.

[35] Mpundu-Kaambwa C, Chen G, Russo R, Stevens K, Petersen KD, Ratcliffe J (2017). Mapping CHU9D Utility Scores from the PedsQLTM 4.0 SF-15. PharmacoEconomics..

[36] Abdin E, Chong SA, Seow E, Verma S, Tan KB, Subramaniam M (2019). Mapping the Positive and Negative Syndrome Scale scores to EQ-5D-5 L and SF-6D utility scores in patients with schizophrenia. Qual Life Res.

[37] Lamu AN, Olsen JA (2018). Testing alternative regression models to predict utilities: mapping the QLQ-C30 onto the EQ-5D-5 L and the SF-6D. Qual Life Res.

[38] Royston P, Sauerbrei W (2007). Multivariable modeling with cubic regression splines: a principled approach. Stata J.

[39] Chen G, Garcia-Gordillo MA, Collado-Mateo D, del Pozo-Cruz B, Adsuar JC, Cordero-Ferrera JM (2018). Converting Parkinson-Specific Scores into Health State Utilities to Assess Cost-Utility Analysis. Patient.

[40] Kaambwa B, Chen G, Ratcliffe J, Iezzi A, Maxwell A, Richardson J. Mapping between the Sydney Asthma Quality of Life Questionnaire (AQLQ-S) and five multi-attribute utility instruments (MAUIs). Pharmacoeconomics. 2017;35(1):111–24. 10.1007/s40273-016-0446-4 27557995

[41] Sopina E, Chenoweth L, Luckett T, Agar M, Luscombe GM, Davidson PM, Pond CD, Phillips J, Goodall S. Health-related quality of life in people with advanced dementia: a comparison of EQ-5D-5L and QUALID instruments. Qual Life Res. 2019;28(1):121–9. 10.1007/s11136-018-1987-0 30187395

[42] Baetschmann G, Staub KE, Winkelmann R. Consistent estimation of the fixed effects ordered logit model. J Royal Stat Soc: Series A (Statistics in Society). 2015;178(3):685–703.

[43] Hyndman RJ, Koehler AB (2006). Another look at measures of forecast accuracy. Int J Forecasting..

[44] Wong CK, Lam CL, Rowen D, McGhee SM, Ma K-P, Law W-L (2012). Mapping the functional assessment of cancer therapy-general or-colorectal to SF-6D in Chinese patients with colorectal neoplasm. Value Health..

[45] Wu EQ, Mulani P, Farrell MH, Sleep D (2007). Mapping FACT-P and EORTC QLQ-C30 to patient health status measured by EQ-5D in metastatic hormone-refractory prostate cancer patients. Value Health..

[46] Petrou S, Rivero-Arias O, Dakin H, Longworth L, Oppe M, Froud R (2015). The MAPS Reporting Statement for Studies Mapping onto Generic Preference-Based Outcome Measures: Explanation and Elaboration. PharmacoEconomics..

[47] Fu T-C, Lin Y-C, Chang C-M, Chou W-L, Yuan P-H, Liu M-H (2016). Validation of a new simple scale to measure symptoms in heart failure from traditional Chinese medicine view: a cross-sectional questionnaire study. BMC Complement Altern Med.

[48] Mogle J, Buck H, Zambroski C, Alvaro R, Vellone E (2017). Cross-Validation of the Minnesota Living With Heart Failure Questionnaire. J Nurs Scholar.

[49] Bilbao A, Escobar A, García-Perez L, Navarro G (2016). Quirós RJH, outcomes qol. Minnesota Living Heart Failure Question.

[50] Zahwe M, Isma'eel H, Skouri H, Al-Hajje A, Rachidi S, Tamim H, Noureddine S. Validation of the Arabic Version of the Minnesota Living with Heart Failure Questionnaire. Heart & Lung. 2020;49(1):36–41. 10.1016/j.hrtlng.2019.10.006 31679804

[51] Brazier JE, Yang Y, Tsuchiya A, Rowen DL (2010). A review of studies mapping (or cross walking) non-preference based measures of health to generic preference-based measures. Eur J Health Econ.

[52] Chuang L-H, Whitehead SJ. Mapping for economic evaluation. British medical bulletin. 2012;101(1). 10.1093/bmb/ldr049 22209743

[53] Chen G, Iezzi A, McKie J, Khan MA, Richardson J (2015). Diabetes and quality of life: comparing results from utility instruments and Diabetes-39. Diab Res Clin Pract.

[54] Chen G, Tan JT, Ng K, Iezzi A, Richardson J (2014). Mapping of Incontinence Quality of Life (I-QOL) scores to Assessment of Quality of Life 8D (AQoL-8D) utilities in patients with idiopathic overactive bladder. Health Qual Life Outcomes..

[55] Olsen JA, Lamu AN, Cairns J (2018). In search of a common currency: A comparison of seven EQ-5D-5 L value sets. Health Econ..

[56] Lamu AN, Chen G, Gamst-Klaussen T, Olsen JA (2018). Do country-specific preference weights matter in the choice of mapping algorithms? The case of mapping the Diabetes-39 onto eight country-specific EQ-5D-5 L value sets. Qual Life Res.

[57] Kaambwa B, Smith C, de Lacey S, Ratcliffe J (2018). Does Selecting Covariates Using Factor Analysis in Mapping Algorithms Improve Predictive Accuracy? A Case of Predicting EQ-5D-5 L and SF-6D Utilities from the Women’s Health Questionnaire. Value Health.

